# Applications of Nano-Selenium in the Poultry Industry: An Overview

**DOI:** 10.3390/nano16020142

**Published:** 2026-01-21

**Authors:** Aya Ferroudj, Hassan El-Ramady, József Prokisch

**Affiliations:** 1 Nanofood Laboratory, Department of Animal Husbandry, Institute of Animal Science, Biotechnology and Nature Conservation, Faculty of Agricultural and Food Sciences and Environmental Management, University of Debrecen, 138 Böszörményi Street, 4032 Debrecen, Hungary; 2 Doctoral School of Animal Husbandry, Faculty of Agriculture and Food Sciences, and Environmental Management, University of Debrecen, 4032 Debrecen, Hungary; 3 Soil and Water Department, Faculty of Agriculture, Kafrelsheikh University, Kafr El-Sheikh 33516, Egypt; hassan.elramady@agr.kfs.edu.eg

**Keywords:** selenium, nanoparticles, cancer, glutathione peroxidase, Japanese quails, antioxidant, oxidative stress, growth, poultry industry, feed to food, one health

## Abstract

Nanotechnology has emerged as a transformative tool in animal production, offering novel strategies to enhance productivity, health, and product quality. Among trace elements, selenium (Se) plays an essential role in antioxidant defence, immune regulation, and redox balance through its incorporation into selenoproteins. Selenium nanoparticles (SeNPs), synthesized via chemical, physical, or biological methods, have shown superior bioavailability, stability, and lower toxicity compared to traditional organic and inorganic selenium forms. This review explores the synthesis, physicochemical properties, and metabolic fate of SeNPs, emphasizing their advantages in poultry production systems. In poultry, SeNPs exhibit potent antioxidant and anti-stress effects by enhancing the activity of glutathione peroxidase, superoxide dismutase, and thioredoxin reductase, thereby mitigating lipid peroxidation and oxidative tissue damage. Their immunomodulatory effects are linked to improved lymphocyte proliferation, cytokine regulation, and increased immunoglobulin levels under normal and stress conditions. SeNP supplementation has been associated with enhanced growth performance, feed efficiency, carcass quality, and reproductive outcomes in broilers, layers, and quails. Furthermore, selenium nanoparticles have demonstrated therapeutic potential in preventing or alleviating chronic diseases such as cancer, diabetes, cardiovascular dysfunction, and neurodegenerative disorders. SeNPs also serve as biofortification agents, increasing selenium deposition in poultry meat and eggs, thus improving their nutritional value for human consumption. However, selenium’s narrow safety margin requires careful dose optimization to avoid potential toxicity. This review highlights the multifaceted benefits of selenium nanoparticles in poultry nutrition and health, while underscoring the need for further studies on grey SeNPs, long-term safety, and regulatory frameworks. Integrating SeNPs into poultry production represents a promising strategy to bridge animal health, food security, and public nutrition.

## 1. Introduction

Nanotechnology has emerged as a promising tool in animal production systems, offering innovative solutions to improve both the quantity and quality of animal-derived products [1]. In poultry production, recent advances in nanotechnology have demonstrated improvements in growth performance, feed efficiency, health status, and product quality [2]. Nanoparticles (NPs) possess unique physicochemical properties compared to their bulk counterparts, including a high surface-to-volume ratio, enhanced surface reactivity, improved stability, increased bioactivity and bioavailability, controlled particle size, and the ability for targeted and controlled delivery [3].

Nanoparticles have also shown strong antimicrobial properties and the potential to reduce microbial load in poultry products, thereby offering alternatives to antibiotics and contributing to the control of antibiotic-resistant pathogens of relevance to human health [4,5]. For instance, silver nanoparticles exhibit antibacterial activity through direct interaction with bacterial cell membranes, generation of reactive oxygen species (ROS), release of Ag^+^ ions, penetration into bacterial cells, and subsequent interaction with DNA [6]. Similarly, zinc oxide nanoparticles (ZnONPs) exert antimicrobial effects by inhibiting bacterial glycolysis, disrupting transmembrane proton transport, interfering with DNA replication, and releasing Zn^2+^ ions and ROS [7].

In poultry nutrition, dietary inclusion of nanoparticles has yielded promising outcomes. Supplementation with selenium and silver nanoparticles has been shown to enhance antioxidant status and reduce oxidative stress in broilers [8,9]. Copper nanoparticles improve immune responses and growth performance [10], while nano-iron has been associated with improved hatchability and productive performance [11,12]. Among various poultry species, including chickens, turkeys, and quails, Japanese quail have also been studied for their efficient growth, egg production, and responsiveness to dietary selenium supplementation under different stress conditions [13,14,15,16]. Among trace elements, selenium nanoparticles (SeNPs) have attracted particular attention due to their capacity to improve nutrient utilization, growth performance, reproductive efficiency, and immune response, while exhibiting lower toxicity and higher bioavailability compared to inorganic selenium sources [17,18,19]. To compile the relevant literature for this review, a comprehensive search was conducted using databases including PubMed, ScienceDirect, and Google Scholar. The search terms included combinations of keywords such as “selenium nanoparticles”, “selenium”, “SeNPs”, “poultry”, “broiler chickens”, “hens”, “growth performance”, “oxidative stress”, “immunity”, and “egg quality”. The search covered articles published primarily between 2010 and 2025, with a focus on studies in peer-reviewed journals. Preference was given to research involving in vivo trials in poultry species. Reference lists of key articles were also screened to identify additional relevant studies.

## 2. Selenium Nanoparticles and Their Characterization

Selenium is an essential trace element for living organisms and is widely distributed in various tissues, including the liver, heart, kidneys, and skeletal muscle [20,21,22,23,24,25,26,27]. Selenium plays distinct physiological roles in plants and animals, contributing to improved growth, productivity, and product quality, particularly under stress conditions, as documented in studies on selenium nutrition [28].

**Figure 1:**
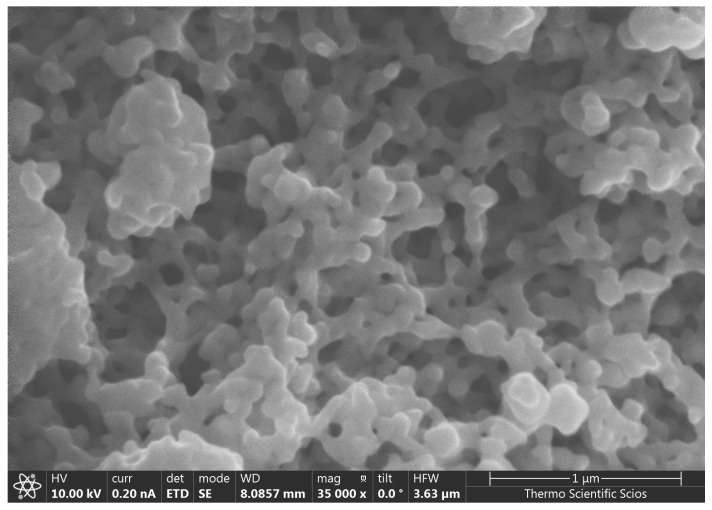
Aggregation of purified red selenium nanoparticles (Se^0^) spherical particles observed using scanning electron microscopy (SEM).

The bioavailability of selenium is influenced by its chemical formula, size, and physicochemical properties, and gastrointestinal absorption remains a major determinant of its biological efficacy. Selenium compounds may transform into insoluble forms as a result of pH changes and microbial activity in the gastrointestinal tract, particularly in ruminants [29]. Elemental selenium nanoparticles (SeNPs) are characterized by low toxicity and zero oxidation state (Se^0^), with particle diameters typically ranging from 100 to 500 nm (Figure 1). Despite being poorly soluble, their nanoscale size enhances bioavailability across microorganisms, plants, and animals [30]. The advantages of selenium nanoparticles arise from their small and uniform size, high permeability, increased stability, and resistance to oxidative and enzymatic degradation, resulting in prolonged residence time and improved biological efficacy [29]. Consequently, the physical form of selenium strongly determines its bioavailability and physiological impact [31].

The chemical form of selenium governs both its bioavailability and toxicity [32]. Inorganic selenium exists primarily as selenate (SeO_4_^2−^) and selenite (SeO_3_^2−^), with selenate generally exhibiting higher toxicity [30]. Regardless of their initial form—selenate, selenite, selenomethionine (SeMet), and selenocysteine (SeCys)—organic selenium compounds are ultimately converted to hydrogen selenide (H_2_Se), which serves as a central intermediate for selenoprotein synthesis or is excreted in urine as selenosugars or via exhalation after methylation [33].

Dietary organic selenium enhances antioxidant capacity by increasing the activity of selenoenzymes that limit peroxide and free radical formation in serum, liver, and peripheral tissues [31,34]. Selenocysteine, the principal biologically active form of selenium, plays a pivotal role in regulating antioxidant defence systems [20]. Common organic selenium sources include selenium yeast, selenomethionine (SeMet), and the hydroxy-analogue of selenomethionine (OH-SeMet), a more stable and highly concentrated source [35].

**Figure 2:**
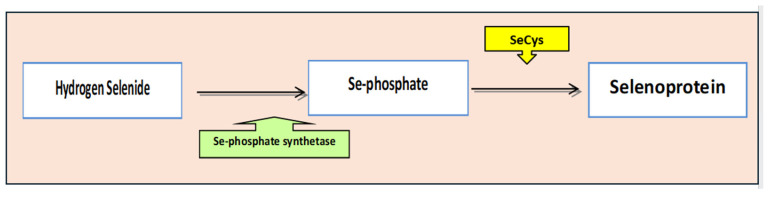
Chemical and biochemical conversion of selenium species from elemental Se to H_2_Se and subsequent incorporation into selenoproteins.

Elemental selenium provides additional physiological benefits through its conversion to hydrogen selenide, an endogenous gasotransmitter involved in immune, endocrine, cardiovascular, and metabolic regulation. These effects are mediated through the incorporation of selenium into redox-active enzymes (selenoproteins) and the maintenance of cellular redox homeostasis (Figure 2) [36,37].

Selenium nanoparticles possess distinctive physicochemical characteristics, including a large surface area, enhanced catalytic efficiency, high adsorption capacity, and reduced toxicity [3]. Both selenium nanoparticles and organic selenium sources exert beneficial effects on poultry health by upregulating selenoprotein expression and activity [38]. Their high bioavailability, combined with relatively low toxicity, makes SeNPs promising alternatives to conventional selenium supplements; however, exceeding physiological selenium requirements remains hazardous and may induce toxicity [17,29,38].

Selenium nanoparticles can be synthesized using chemical, physical, or biological methods. Chemical synthesis commonly involves the reduction of selenious acid (H_2_SeO_3_) or selenite ions using reducing agents such as ascorbic acid in aqueous solutions, with the characteristic colour change to red indicating nanoparticle formation [29]. Physical methods include laser ablation and ultrasonic fragmentation using selenium-containing substrates [29,39]. Biological synthesis exploits the ability of plants and microorganisms to convert selenium ions into elemental nanoparticles, yielding uniformly spherical and biocompatible SeNPs with minimal toxicity [29,32].

The probiotic bacteria present in yoghurt can produce elemental SeNPs (50–500 nm) through intracellular detoxification of high selenite concentrations, reducing selenium ions to elemental nanospheres [32]. Such biological methods are considered environmentally friendly and suitable for nutritional applications [29,32].

Approximately 80% of dietary selenium absorption occurs in the duodenum, with organic forms generally exhibiting higher absorption efficiency than inorganic forms. Selenium enters enterocytes through distinct mechanisms: selenite diffuses passively across cell membranes, selenate is absorbed via sodium-dependent transporters, and selenomethionine utilizes amino acid transport pathways similar to methionine [20,29]. Once absorbed, selenium is transported in the bloodstream bound to plasma proteins, mainly albumin, and delivered to the liver, where selenium metabolism occurs. It is subsequently converted to hydrogen selenide in the liver and then incorporated into selenoprotein P, which transports selenium via the bloodstream to peripheral tissues, where it is taken up through specific receptors such as LRP (low-density lipoprotein receptor-related protein), or excreted following methylation [20,29,40].

## 3. Poultry Stressors and Oxidative Impact

Stress has become a critical challenge in modern poultry production, affecting physiological functions, immune competence, and overall productivity. Like other living systems—including plants and microorganisms—poultry is highly susceptible to stressors that compromise homeostasis. These stressors originate from environmental, nutritional, biological, and managerial factors, and their cumulative impact can lead to oxidative stress, reduced growth performance, and diminished product quality. Environmental stressors, particularly extreme temperatures, are among the most detrimental. Poultry species lack sweat glands and have a limited ability to dissipate heat, making them vulnerable to heat stress. Exposure to high or low ambient temperatures triggers the excessive production of reactive oxygen species (ROS), which damage cellular components, reduce feed efficiency, and impair immune function. Heat stress, in particular, has been linked to decreased meat quality, myoglobin oxidation, reduced shelf life, and poor laying performance [41,42]. Nutritional stressors arise from unbalanced diets, deficiencies in essential nutrients (such as selenium, zinc, vitamins A and E, and amino acids), or the presence of contaminated feed ingredients. Mycotoxins, including aflatoxins, ochratoxins, and fumonisins produced by fungi in poorly stored feed, are well-known contributors to oxidative damage and immunosuppression [43,44]. Selenium (Se) is particularly vital for antioxidant defence, and its deficiency significantly increases susceptibility to oxidative stress. However, industry stakeholders now face the challenge of identifying sustainable, safe, and bioavailable Se sources that can ensure long-term profitability and productivity. Biological stressors include viral (e.g., avian influenza), bacterial, and fungal infections, all of which trigger ROS accumulation and inflammation. These pathogens not only impair gut health and nutrient absorption but also suppress antioxidant enzyme systems [45]. Managerial stressors—such as overcrowding, poor ventilation, transportation stress, mechanical vibrations, and inadequate lighting—contribute to chronic stress that weakens immunity and predisposes birds to injury and disease [46,47,48]. These conditions reduce growth rates and feed efficiency and ultimately diminish the economic viability of poultry operations.

## 4. Main Applications of Selenium Nanoparticles in Poultry

Adequate selenium (Se) nutrition is essential in poultry to ensure optimal immune function and overall physiological performance. Appropriate dietary selenium levels reduce the risk of several health disorders, including oxidative stress, muscular dystrophy, cardiovascular dysfunction, cystic fibrosis, and inflammatory joint conditions [49]. Cellular structures and macromolecules are highly sensitive to oxidative stress, which disrupts cellular homeostasis. Oxidative stress leads to the excessive generation of reactive oxygen species (ROS), including hydroxyl radicals, superoxide anions, and hydrogen peroxide, thereby promoting apoptotic pathways at the cellular level [50].

Major physiological and metabolic sources of oxidative stress include mitochondrial respiration, phagocytic and inflammatory responses of the innate immune system, xenobiotic metabolism, detoxification processes, prostanoid synthesis, redox-active transition metals (iron and copper), and exposure to high concentrations of oxygen and polyunsaturated fatty acids [51,52]. The expression of selenoproteins is primarily regulated by dietary selenium availability and cellular redox status [53]. During mitochondrial respiration, molecular oxygen is partially reduced to reactive oxygen species, while phagocytes deliberately produce peroxides as antimicrobial agents. However, excessive peroxide generation may cause collateral damage to host tissues. Moreover, detoxification by superoxide dismutase (SOD) is incomplete, as it results in hydrogen peroxide formation, which must be further reduced to water by catalase or glutathione peroxidase (GPx) [51]. Reactive oxygen species target critical biological macromolecules, including proteins, lipids, and DNA, thereby impairing essential cellular and tissue functions [35]. In poultry production, oxidative stress is exacerbated by environmental stressors such as temperature fluctuations, immunosuppression, and mycotoxin exposure, negatively affecting meat and egg quality. Selenium supplementation in poultry diets mitigates the deleterious effects of ROS by enhancing antioxidant defence systems [25,54,55]. Conversely, selenium deficiency compromises productivity, weakens immune responses, and increases chick mortality [25,56,57]. Malondialdehyde (MDA), the terminal product of polyunsaturated fatty acid peroxidation, is widely used as a biomarker of oxidative stress and lipid peroxidation in poultry tissues [31,58,59].

### 4.1. Selenium and Nano Selenium Mediated Immune Enhancement in Poultry

The immunomodulatory effects of selenium reported in Table 1 are primarily mediated through selenium-dependent proteins and antioxidant regulation. Selenium is incorporated into key selenoproteins, including glutathione peroxidases, thioredoxin reductases, and selenoprotein P, which maintain intracellular redox balance and support both innate and adaptive immune responses [35,60]. By strengthening enzymatic and non-enzymatic antioxidant defences, selenium protects immune cells from oxidative damage, reduces lipid peroxidation, and enhances immune resilience under normal and stress conditions [54].

In addition to its antioxidant role, selenium contributes directly to antiviral and cell-mediated immunity by upregulating interferon-related genes, enhancing lymphocyte proliferation, and improving antibody production following vaccination [61,62,63]. Selenium supplementation has also been shown to modulate cytokine responses under stress, reducing pro-inflammatory signalling and supporting immune stability during heat stress [54,62,63]. Collectively, these mechanisms explain the improved immune performance and disease resistance observed in selenium-supplemented poultry.

Importantly, the magnitude of these effects is strongly dependent on selenium source and dosage. Organic selenium and nano-selenium consistently outperform inorganic sodium selenite, with optimal responses generally reported at dietary levels of 0.15–0.5 mg/kg [64,65]. However, the available evidence is derived almost exclusively from studies using red, amorphous selenium nanoparticles, leaving unresolved whether grey selenium nanoparticles exhibit comparable bioavailability and immunological activity.

**Table 1:** Biological effects of selenium nanoparticles in poultry: antioxidant-immunity modulation.

Se Form	Species/Model	Dose (mg/kg Diet)	Antioxidant Effects	Immune Effects	Reference
Various Se forms (review)	Poultry and mammals	Not specified	Selenium regulates cellular redox homeostasis through the activity of selenoproteins	Selenium participates in the regulation of both innate and adaptive immune responses	[60]
Various Se forms (review)	Poultry	Not specified	Selenium supplementation enhances antioxidant protection, particularly under stress conditions	Improved immune resilience during infection and environmental stress	[66]
Organic Se	Broilers	0–0.3	Dietary organic selenium increased glutathione peroxidase activity and reduced lipid peroxidation	Selenium supplementation stimulated lymphocyte proliferation in a dose-dependent manner	[67]
Organic Se (yeast enriched) vs. Na_2_SeO_3_	Broilers	0.3	Organic selenium improved antioxidant indices compared with inorganic selenium	Improved physiological condition and immune status	[68]
Nano-Se	Broilers	0.5	Selenium nanoparticles enhanced antioxidant enzyme activities and reduced oxidative stress	Increased antibody production following supplementation	[61]
Nano-Se (red)	Broilers	0.2–0.6	Selenium nanoparticles reduced malondialdehyde concentrations and increased glutathione and glutathione peroxidase levels	Not reported	[69]
Nano-Se vs. Na_2_SeO_3_	Broilers	0.15–1.2	Selenium nanoparticles resulted in higher glutathione concentrations than sodium selenite	Not reported	[64]
Nano-Se vs. Na_2_SeO_3_	Japanese quail	0.1–0.2	Selenium nanoparticles increased glutathione peroxidase activity and reduced lipid peroxidation	Selenium nanoparticles improved measured immune indices	[70]
Nano-Se	Broilers	~0.3	Selenium nanoparticles increased glutathione peroxidase and superoxide dismutase activities and reduced lipid peroxidation	Not reported	[31]
Nano-Se	Broilers	~0.9	Selenium nanoparticles improved the overall oxidative status	Enhanced immune responses and modulation of gut health	[71]
Green Nano-Se vs. Na_2_SeO_3_	Broilers	~0.3	Selenium nanoparticles reduced oxidative damage and increased antioxidant capacity	Improved immune competence	[72]
Nano-Se (review)	Poultry	Not specified	Selenium nanoparticles contribute to improved oxidative balance	Selenium nanoparticles enhance immune function in a form-dependent manner	[65]
Functionalized SeNPs	In vitro	Not specified	Modulation of oxidative stress was dependent on nanoparticle surface chemistry	Not reported	[73]
Organic and inorganic Se	Chickens	Nutritional	Not reported	Selenium supplementation increased antibody levels and reduced viral shedding	[63]
PVP-SeNPs	In vitro bacteria	MIC ≈ 0.3 µg/mL	Not reported	Strong antibacterial activity against *Staphylococcus aureus*, *Bacillus cereus*, *Klebsiella pneumoniae*, *Escherichia coli*, and *Pseudomonas aeruginosa,* and antitumor activity against MRC-5 carcinoma cell line	[62]
SeNPs	In vitro pathogens	0.5–100 µg/mL	Not reported	*Listeria monocytogenes*, *Staphylococcus aureus*, *Staphylococcus epidermidis*, *Vibrio alginolyticus*, *Salmonella enterica*, SeNPs display strong antifungal activity against *Candida albicans*.	[74]
Nano-Se, Met-Se, Cys-Se, Na_2_SeO_3_ (review)	Poultry	Various	Selenium nanoparticles and organic selenium sources showed stronger antioxidant effects than inorganic selenium	Selenium nanoparticles and organic selenium sources enhanced immune responses	[38]

### 4.2. Selenium as an Anti-Stress and Antioxidant Agent

Antioxidant protection is achieved through the neutralization and removal of reactive oxygen species (ROS) via several complementary mechanisms. Antioxidants can be broadly classified into fat-soluble components, such as vitamin E, carotenoids, and coenzyme Q, and water-soluble components, including vitamin C, glutathione, thioredoxin, carnitine, and taurine. In addition, antioxidant defence relies on a complex enzymatic system that includes glutathione peroxidases (GPx1–4, 6), multiple selenoproteins (I, M, K, H, N, O, P, V, R, S, and T), glutathione transferase, glutathione reductase, superoxide dismutase (SOD), and thioredoxin reductases [20,58,75]. Glutathione represents a major non-enzymatic intracellular antioxidant component and plays a central role in maintaining redox homeostasis [31]. The primary enzymatic defence against oxidative stress is mainly mediated by GPx and SOD, whose activities are functionally interconnected [31,38]. Glutathione peroxidase (GPx) is a selenium-dependent enzyme that is activated under oxidative stress conditions arising from β-oxidation and peroxide generation, which can disrupt normal cellular functions. GPx protects cells by reducing peroxides and limiting the activity of enzymes responsible for the generation of toxic free radicals [76]. GPx activity is reported to be highest in the liver and kidneys, lower in plasma and red blood cells, and lowest in thigh and pectoral muscles [20,34].

Selenoproteins are classified into two major groups: those that maintain essential biological functions under normal conditions and stress-responsive selenoproteins that are induced during oxidative stress or selenium deficiency [20]. In chickens, 25 genes encoding selenoproteins have been identified [77,78,79]. The synthesis and activity of these selenoproteins strongly depend on selenium availability and physiological stress status. While some selenoproteins show constitutive low-level expression to support basal cellular functions, the majority exhibit selenium-dependent regulation linked to their biological activity [20,35,51,80].

Dietary supplementation with selenium nanoparticles (SeNPs) has been shown to enhance both the expression and activity of selenoenzymes in living organisms [31,34] (Table 2). The active sites of selenoproteins are selenium-specific, enabling selenium to function as an essential cofactor responsible for enzyme activation and biological function [39]. Optimal selenium nanoparticle doses required for the activation of GPx1, GPx4, and TxR have been demonstrated previously [34]. Furthermore, SeNP supplementation enhances GPx and SOD activities, supports glutathione redox cycling, and prevents excessive accumulation of malondialdehyde (MDA), a key marker of lipid peroxidation [38,76].

As a preventive strategy, cells store selenium intracellularly in the form of selenoproteins to ensure availability during periods of selenium deficiency or physiological stress [20,29,81]. Skeletal muscle serves as a major selenium reservoir, which can be mobilized under stress to maintain the basal expression of essential selenoproteins [20,35,51,80].

Biological systems operate through three distinct levels of antioxidant protection. The first level involves TxR, selenoproteins R and W, and cellular detoxification by SOD, which converts superoxide radicals into hydrogen peroxide (H_2_O_2_). Although H_2_O_2_ is potentially harmful, it is subsequently reduced to water by catalase and glutathione reductase [75,82]. This level also includes the sequestration of free transition metals by specific binding proteins to reduce oxidative reactions [20]. Additionally, carnitine, taurine, and coenzyme Q play crucial roles in maintaining mitochondrial functional integrity [82,83].

The second defence level relies primarily on the glutathione system, comprising reduced glutathione (GSH), GPx, glutathione reductase, and glutathione disulfide reductase, alongside ascorbic acid, vitamin E, carotenoids, thioredoxin, thioredoxin reductase, and peroxiredoxins [75]. Within this system, oxidized vitamin E is regenerated to its reduced, biologically active form through reactions involving vitamin C and the thioredoxin system, with NADPH supplied by the pentose phosphate pathway [75].

The third and final level consists of cellular repair mechanisms, including heat shock proteins (HSPs), methionine sulfoxide reductase, phospholipases, and DNA repair enzymes, which collectively restore macromolecular integrity following oxidative damage [84,85].

**Table 2:** Effect of selenium nanoparticles on poultry growth performance and antioxidant biomarkers.

Bird	Body Weight (g)	Food Conversion Ratio (g/g)	Feed Intake (g/d)	GPx (mg/dL)	MDA (nmol/mL)	SOD (U/mL)	[SeNPs] (mg/kg)	Reference
Arbour broiler	368.30	1.54	557	NA	7.4	251.4	0	[86]
	387.40	1.48	580.9	NA	27.4	273.8	100	
Ross broiler	364.20	1.76	643.2	NA	8.2	256.3	0	
	412.80	1.40	543	NA	30.3	275.1	100	
Broiler Japanese quail	4.43	6.37	28.18	74.60	3.86	NA	0	[87]
	3.91	6.78	26.46	60.90	4.88	NA	0.2	
	3.95	6.77	26.71	66.60	4.54	NA	0.5	
Japanese quail	178.50	3.49	18.9	0.13	0.33	0.12	0	[69]
	198.10	2.81	17.1	0.30	0.22	0.29	0.4	
Broiler Arbour Acres	47.30	1.64	77.3	1.18	3.90	146	0	[31]
	48.10	1.61	77.2	1.41	3.20	171	0.3	
	48.20	1.62	77.9	1.39	3.30	156	0.5	
Hen	NA	NA	131.09	2817.49	12.86	67.39	0	[88]
	NA	NA	131.41	3437.77	9.8	55.02	180	

GPx: Glutathione peroxidase, MDA: malondialdehyde, SOD: superoxide dismutase, NA = not available.

### 4.3. Production Performance with Selenium Nanoparticle Supplies in Poultry

In modern poultry production systems, birds are routinely exposed to various stressors, which can be broadly classified as environmental, nutritional, biological, and technological in origin. These include heat stress, pathogen exposure, mycotoxins, and intensive housing conditions, all of which can disrupt physiological homeostasis and trigger oxidative stress. The overproduction of reactive oxygen species (ROS) under these conditions is now recognized as a central mechanism impairing poultry performance [20,52,84,89]. Among these stressors, ambient temperature plays a particularly influential role. The thermoneutral zone for poultry generally ranges from 25 °C to 36 °C, with optimal egg production occurring near 23.8 °C [90,91]. Elevated temperatures reduce feed intake and increase water consumption, ultimately compromising growth rate, feed efficiency, egg quality, and immune function [92,93]. Among poultry species, Japanese quail (Coturnix japonica) have received increasing attention due to their rapid growth, early sexual maturity, high egg-laying capacity (up to 320 eggs per cycle), and efficient feed conversion, despite their lower daily intake (20–25 g/bird) [13,14,15,16]. Quails also require less housing space and exhibit greater resistance to common poultry diseases, making them a cost-effective alternative to chickens, especially in resource-limited settings [16,94,95,96]. The nutritional value and market acceptance of quail eggs have contributed to the growth of quail farming in recent years [15,16,97]. However, quail and their eggs remain vulnerable to environmental stressors—particularly temperature fluctuations during storage—which can negatively affect productivity, egg quality, and microbial safety [16,20,52,80,85,89,98].

To reduce oxidative stress and maintain productivity, selenium nanoparticles (SeNPs) have emerged as effective nutritional additives. Compared to inorganic selenium sources such as sodium selenite, SeNPs offer higher bioavailability, lower toxicity, and enhanced tissue deposition [99]. Studies have shown that dietary inclusion of SeNPs at 0.15–0.5 mg/kg significantly improves body weight gain, feed conversion ratio (FCR), carcass traits, and antioxidant capacity in broilers and native breeds like Guangxi yellow chickens [34,64,100]. Additionally, SeNP supplementation at 0.3 mg/kg has been linked to increased glutathione peroxidase activity and immunoglobulin M (IgM) levels, enhancing both antioxidant defences and humoral immunity [31]. For instance, SeNP supplementation reduced the incidence of fatty liver syndrome in broilers and suppressed pathogenic microorganisms, highlighting its dual role in metabolic health and disease resistance [101].

Further evidence supports these outcomes across various forms and synthesis methods. Supplementation with green-synthesized SeNPs improved growth performance, carcass yield, and serum antioxidant markers in broilers [102,103], while biogenic SeNPs produced using *Bacillus subtilis* enhanced gut microbiota and immune responses [104]. The biologically synthesized SeNPs also contributed to improved carcass traits and economic efficiency [105]. Notably, chitosan-loaded SeNPs significantly improved intestinal morphology and nutrient absorption, reinforcing growth benefits at the gut level [106]. In addition, SeNPs have been shown to enhance the quality of frozen broiler meat more effectively than either inorganic or organic selenium sources, likely due to improved oxidative stability and preservation of muscle integrity [107].

Parallel benefits have been observed in laying hens. Shu et al. [108] reported that chitosan SeNP supplementation enhanced egg quality, shell strength, and selenium deposition in ageing hens. Gebriel et al. [109] found similar improvements in laying rate, oxidative resistance, and tissue selenium levels. Additionally, Radwan et al. [110] reported increased egg selenium content and improved lipid stability, extending shelf life. Malik et al. [111] corroborated these findings, noting enhanced immune response, yolk selenium levels, and overall laying performance. Collectively, these studies affirm that SeNPs can improve productivity, oxidative stability, and immune regulation more effectively than conventional selenium sources.

However, selenium’s narrow margin between nutritional requirement and toxicity necessitates precise dietary control. While SeNPs are generally less toxic than inorganic forms, excessive selenium intake, especially above 0.5 mg/kg, may result in tissue accumulation and adverse physiological effects. Clinical signs of selenosis in poultry can include reduced feed intake, feather loss, organ lesions, and even mortality in severe cases [112]. Although SeNPs exhibit a higher safety threshold due to their controlled release and lower pro-oxidant potential, responsible dosing remains essential. Regulatory bodies such as the Association of American Feed Control Officials (AAFCO) recommend a maximum inclusion level of 0.5 mg/kg selenium in poultry diets to prevent toxicity [113]. Based on available studies, SeNPs have shown optimal efficacy and safety at a dietary dose of 0.3 mg/kg in broilers [34].

Beyond growth performance, SeNPs also play a key role in enhancing egg production and reproductive efficiency (Table 3). They stimulate epithelial secretions involved in eggshell membrane formation, thereby improving shell thickness and structural integrity. During embryogenesis, SeNPs enhance antioxidant protection by reducing lipid peroxidation and supporting yolk stability, which reduces the risk of malformations and embryonic mortality [25,114]. Selenium is efficiently transferred from maternal diets to eggs and embryos, where it supports endogenous antioxidant enzyme systems [52,75,80,82,83,85,115,116]. SeNP supplementation has also been shown to increase laying rate, egg mass, albumen quality, ovulation rate, hatchability, and Haugh unit score while reducing embryonic mortality [65,117]. These benefits are partly attributed to selenium’s antioxidant and antimicrobial functions, which limit bacterial penetration through the eggshell and preserve internal egg quality [118]. However, egg quality remains vulnerable to post-laying environmental conditions. Elevated storage temperatures promote microbial growth, including moulds and Gram-negative bacteria, leading to albumen degradation and reduced shelf life [25,98]. Refrigeration at 4 °C for up to 120 days has been shown to maintain microbial safety and egg integrity, preserving the benefits conferred by selenium-enriched diets [16].

### 4.4. Medical Attribute

**Figure 3:**
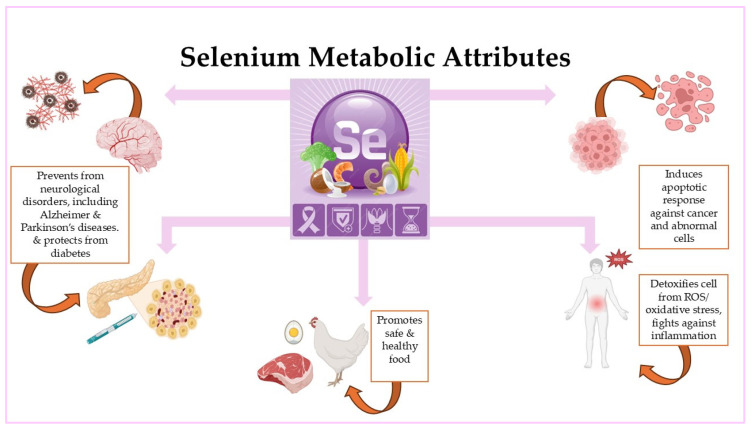
Selenium metabolic and medical attributions.

Selenium plays a crucial role in numerous aspects of human and animal health, including inflammation, cancer, immune function, and diabetes [124] (Figure 3). It exhibits a dual role in glucose metabolism by improving insulin synthesis and secretion and by exerting insulin-mimetic effects that enhance intracellular glucose uptake [124]. High doses of inorganic and organic selenium have been administered to alleviate symptoms in experimental models of both type 1 and type 2 diabetes. Furthermore, improvements in metabolic status have been reported in patients with type 2 diabetes (T2D) following dietary selenium supplementation. However, several livestock studies indicate that excessive selenium intake may exert diabetogenic effects, highlighting the narrow therapeutic window of selenium supplementation [124].

Selenium nanoparticles (SeNPs) have demonstrated efficacy in inhibiting cell proliferation in high-grade serous ovarian cancer cells through multiple biological mechanisms. Previous studies have shown that selenium can suppress cancer cell growth via autophagy in colorectal cancer, apoptosis in skin, breast, and liver cancers, and ROS-mediated necrosis in prostate cancer [125]. Selenium enhances cellular detoxification against reactive oxygen species (ROS) in most tissues; excessive ROS production induces oxidative stress, leading to DNA damage and apoptosis. Selenium deficiency may prolong oxidative stress and increase cancer risk. In several lung cancer cell lines, certain inorganic selenium compounds have been found to be more effective than conventional cytotoxic drugs through modulation of the thioredoxin system. Another key mediator of selenium activity is selenoprotein P (SelP), which serves a dual function as both an antioxidant and a selenium transport protein with anticarcinogenic properties. Downregulation of SelP expression commonly leads to increased oxidative stress and altered glutathione peroxidase (GPx) activity in tumour tissues [58].

#### 4.4.1. Selenium and Cardiovascular Health

Cardiovascular diseases are associated with hyperlipidaemia, increased blood viscosity, atherosclerosis, hypertension, and elevated mortality rates, particularly among elderly populations. The beneficial effects of selenium on cardiovascular health have been well documented [126]. Selenoenzymes such as thioredoxin reductase (TrxR) and GPx play a protective role in the vascular endothelium by reducing oxidative damage, inhibiting platelet aggregation, and attenuating inflammation. GPx4 contributes to the reduction in phospholipid hydroperoxides and cholesterol ester hydroperoxides in lipoproteins, thereby limiting oxidized low-density lipoprotein (LDL) accumulation in vascular walls and reducing thrombotic risk. Selenium deficiency leads to reduced GPx activity and elevated lipid hydroperoxide levels, which inhibit prostacyclin synthase, an enzyme essential for prostacyclin production. A decline in prostacyclin—a potent vasodilator and inhibitor of platelet aggregation—favours increased thromboxane synthesis, promoting platelet aggregation and increasing cardiovascular risk [58].

#### 4.4.2. Selenium and Neurodegenerative Disorders

Low selenium (Se) levels have been associated with several neurological disorders, including Alzheimer’s disease, Parkinson’s disease, epilepsy, and schizophrenia. Deficiency of selenoprotein P reduces glutathione peroxidase (GPx) and thioredoxin reductase (TrxR) activities and decreases selenium availability in the brain, contributing to oxidative damage and motor dysfunction [58]. Evidence indicates a protective role of selenium against Alzheimer’s disease. Selenium supplementation has been shown to prevent and attenuate Alzheimer’s pathology by enhancing antioxidant defences and reducing oxidative stress [127]. Consistently, decreased selenium levels have been linked to the onset and progression of Alzheimer’s disease [128]. The relationship between selenium status and Parkinson’s disease appears dose-dependent. While excessive selenium exposure may increase Parkinson’s disease risk [129], higher physiological blood selenium levels have been associated with reduced disease prevalence [130]. This duality highlights the narrow optimal range of selenium for neuroprotection. Adequate glutathione levels play a key role in mitigating oxidative stress and supporting neuronal recovery in Parkinson’s disease [131], underscoring the importance of selenium-dependent antioxidant systems.

#### 4.4.3. Selenium and Cancer

Selenium (Se) is a cofactor incorporated into several selenoproteins, such as glutathione peroxidase (GSH-Px), thioredoxin reductase (TrxR), and selenoprotein P1 [132]; those are the most well-known antioxidant enzymes in living systems [133,134]. All selenium compounds might contribute to selenoenzyme biosynthesis, and several studies indicate that the bioavailability of Se increases selenoprotein (SelP) expression in vivo and in vitro [132,135,136]. Different metabolism pathways are involved to produce a useful intermediate form of Se, like selenide, which is inserted as a Se-amino acid (SeCys) in the peptide chain during translation of mRNA due to its unique codon [132,134].

Selenoproteins catalyze disulfide bonds between the protein macromolecule to maintain their functional structures [137,138]. Selenium potentiates the ability to diminish oxidative cell status by integrating into selenoproteins, which is the key behind its anticancer and antioxidant impacts [139]. Methyl-selenol (CH_3_SeH) is one of the intermediate metabolites of selenium that has a powerful anticancer effect in case of over-nourishment of that element, by detoxifying cells from carcinogens and inducing caspases, and limits tumour cell proliferation [134]. On the other hand, inorganic forms such as selenite enhance immune system response at low doses by favouring natural killers (NK) and lymphocytes production [134]. Selenoenzymes exhibit distinct activities in carcinoma cases; thus, the molecular expression of these proteins is altered. Such an example is GPx4, which is shown to have a therapeutic and biomarker behaviour in lymphatic system cancer [140]. Their high expression in sane tissues provides cancer protection, or, in the case of TrxR, can lead to tumour cells ’apoptosis [141,142]. Selenium has two opposite effects: an anticancer effect by preventing malignancy in several organs, and it may also induce tumour development. Its different compounds have a remarkable impact to protect from hepatic, colorectal, and breast carcinoma by favouring (SeP.) expression and protecting DNA from abnormal cell damage [143]. In addition, Se deficiency or over-exposure can produce harmful responses. In case of high uptake, the epidemiologic cancer risk is significantly increased and results in keratinocyte carcinoma, squamous cell cancer, and hyperglycaemia (Diabete-2) [139,144]. Selenium deficiency promotes cardiovascular disorders and neuropathy because of the oxidative stress and low rate of selenoenzymes, and might influence the reproductive capacity for both genders and general immune system weakness [143,145]. Redox-active selenium species conversion to elemental (Se^0^) is due to their pro-oxidant properties, which can generate more free radicals that promote mitochondrial apoptosis or cell necrosis in very high amounts [132,146]. As a positive side, these pro-oxidant compounds can be used as a chemotherapeutic cure as the cancer cells have a high affinity to them; several studies have reported the positive impact of Se in preventing prostate cancer and reducing side effects of conventional radio-chemotherapies. Typically, tumour cells produce high amounts of reactive oxygen species (ROS), but they have low resilience to them, so the active Se compounds can induce oxidative stress, which leads to the apoptosis of these abnormal cells [133,147]. Selenium acts as chemoprotection in the early stage of tumour progression by inhibiting fat peroxidation and preventing DNA adducts produced by carcinogens such as (7,12-dimethylbenz(a) anthracene) “DMBA” [133]. The chemical forms of selenium might also interact differently with the tumour suppressor gene P53 and induce different responses [148]. So, they may stimulate DNA repair or cell apoptosis. Even serum content of Se can lead to a modulation of chemotherapy and radiotherapy drugs [149,150]. A recent study demonstrates that in vitro SeNPs have the aptitude to distinguish between normal and cancer cells and promote cell death via high cytotoxicity in tumours [151]. The key behind the impressive effect of nano-sized selenium to fight against cancer development lies in its multiple mechanisms of action, such as inducing energy shortages in abnormal cells—reductions in ATP, ADP, and NAD^+^, and blockage of cellular respiratory pathways [147,152]. Supplementation of chemically produced SeNPs leads to elevated cysteine and fructose levels in cancer cells, imitating the effects of hypoxia and oxidative stress, and suppresses their uncontrolled multiplication while arresting the cellular cycles [147,153]. In addition, the nanoparticles of selenium restrict the essential amino acids and fatty acids necessary for cancer progression [147,152].

#### 4.4.4. Selenium and Diabetes

Selenium is an indispensable element for the proper functioning of selenoproteins in several organs and tissues of the living body. The rate of alteration of selenium in a population can induce numerous medical issues. Diabetes is one of the most common illnesses nowadays. The relation between diabetes and Se abundance is a critical multifaceted relationship, depending on several conditions such as gender, age, and nutritional status [154,155,156]. Many studies demonstrate that high uptake levels of selenium can lead to the high expression of selenoprotein P (Sep P) and serum Se content, which may lead to insulin resistance and diabetes type 2 (T2D). On the other hand, the mechanism of Sep (P) in influencing glucose metabolism involves the inactivation of adenosine monophosphate kinase AMPk, which is responsible for insulin sensitivity and fatty acid oxidation, while also activating the expression of gluconeogenic enzymes, thereby explaining the hyperglycaemia observed [155,156]. In contrast, a new study revealed that there was no correlation between Se concentrations in serum and glycemia parameters [157]. Additionally, Se is known as a protective factor against hypertensive nephropathy and decreases the risk of diabetes related to it [158]. Ref. Ogawa-Wong et al. [159] reported that high exposure to Se increases T2D risk in males, whereas females are not affected. This poses the hypothesis that selenium–glucose homeostasis is regulated in a sex-dependent manner. High Se uptake increases the expression of several selenoproteins, such as Sep P, GPx1, and Sep S, and leads to over-scavenging of intercellular ROS, which impairs insulin receptors and downstream signalling, ultimately leading to hyperinsulinemia and glucose intolerance [144,160,161,162]. On the other hand, refs. [156,163,164,165] suggested that large quantities of antioxidant biomarkers may be a consequence rather than a cause of T2D, and it may signify hyperglycaemia that proposes an insulin mimetic effect for selenium and SeNPs and may be an antidiabetic element at certain levels. Biosynthesized crystallin SeNPs can prevent tissue damage induced by diabetes via the enhancement of antioxidant activities in organs such testis [166]. Ref. [167] demonstrated that nanoparticles of selenium stabilized with polysaccharides had antidiabetic potential in diabetic mice; additionally, these NPs of selenium could prevent the offspring of gestational diabetic mothers from developing diabetes [168]. Moreover, the combination of SeNPs with plasma-rich platelets can reduce complications associated with diabetes [169]. Green-synthesized SeNPs with *Moringa oleifera* have a similar impact to insulin by reducing hyperglycaemia and deactivating α-amylase and α-glucosidase [170].

#### 4.4.5. Selenium and Dermatological Conditions

Stress severely compromises skin health by increasing ROS (Reactive Oxygen Species), which drives inflammation, collagen breakdown, and accelerated ageing [171]. Selenium counters this by boosting antioxidant capacity and maintaining thyroid homeostasis. This systemic support facilitates the optimal growth and differentiation of keratinocytes, thereby improving the health and elasticity of the skin, hair, and nails [172]. Selenium, as a mineral cofactor, provides protection to the external layer of the skin from various dermatological disorders like eczema, acne, and psoriasis [173], due to the antimicrobial and anti-inflammatory potential, which promotes wound healing and prevents infection [174]. In addition, selenium exhibits anti-infection activity, protecting against several Gram-positive bacteria such as *Staphylococcus aureus* and *Bacillus cereus*, Gram-negative bacteria such as *Listeria monocytogenes*, and the yeast *Saccharomyces cerevisiae* [73]. In addition to recovering sores, SeNPs regulate the pro-inflammatory cytokines Il-6 and TNF-α by reducing their levels to accelerate repair [175]. Ref. [176] demonstrated that SeNPs at 0.5 (mg·kg^−1^) can accelerate healing by decreasing nitric oxide amounts and protecting injured tissues from oxidative stress and apoptosis, while cicatrization factors such as vascular endothelial growth factor (VEGF) and collagenase I show elevated activity. Additionally, selenium has different forms and ionic states, and each could have its own impact on skin health. For example, selenium sulphide (SeS_2_) reduces the formation of dandruff and other forms of forehead eczema [177]. Ref. [178] has been shown that zinc and copper are effective in cases of severe eczema (vitiligo and alopecia), but selenium has not clearly demonstrated an impact comparable to that of the previously mentioned elements. Ref. [173] demonstrated that selenium deficiency can lead to other specific dermatological illnesses like acne vulgaris, chloric acne, and psoriasis. Although [179] reported that the prevalence of childhood eczema does not show a clear link with Se intake rate, Ref. [180] demonstrated a strong protective effect against sunburn-related skin issues when selenium was administered in combination with probiotics. Therefore, we may say that the correlation between selenium and skin wellness is a complicated relationship. Se confers significant anti-ageing and photoprotective properties to the skin, primarily by acting as a powerful antioxidant and supporting genomic stability. Selenium is incorporated into selenoproteins (including GPx1, TXNRD1, MsrB1, and SelenoP), which are crucial for detoxifying reactive oxygen species (ROS) and facilitating cellular DNA repair [181,182]. This action is vital for maintaining the stability of the entire genome and preventing DNA damage, mutation, and age-related decline. Furthermore, selenium intake, specifically as sodium selenite, has been linked to the superior maintenance of telomere length, a key factor in slowing cellular senescence [181]. At the cellular level, selenium supports epidermal integrity by preserving the longevity of Keratinocyte Stem Cells (KSCs) and enhancing keratinocyte adhesion to the basement membrane via increased collagen and integrin expression. By controlling cell cycle progression through the activation of tumour suppressor pathways (e.g., p53,21,16), selenium prevents premature senescence and supports continuous epidermal renewal [183]. Collectively, these actions underscore selenium’s role in the skin’s self-renewal pathway, its capacity to mitigate the damaging effects of chronic UV radiation exposure, and its overall support for long-term skin health and elasticity [184].

### 4.5. Selenium Nanoparticles Biofortification in Poultry Products for Human Consumption

Poultry products, particularly meat and eggs, represent an efficient and sustainable vehicle for selenium biofortification, offering a practical strategy to enhance human dietary selenium intake. Selenium deficiency remains a global nutritional concern, affecting immune competence, thyroid function, and antioxidant defence in many populations [145]. The introduction of selenium nanoparticles into poultry diets offers a viable solution to this problem via feed-to-food nutrient transfer [135,185]. SeNPs are more bioavailable, less toxic, and better retained in avian tissues than inorganic forms like sodium selenite [186]. This leads to elevated selenium accumulation in muscle, liver, and egg yolk [34,187]. These biofortified products can provide selenium in organic and nano-forms, which are better absorbed by humans than conventional sources [185]. The feed-to-food nutrient transfer approach is a potential solution to the deficiency issue by supplementation of selenium nanoparticles to poultry diets. In addition to boosting selenium content, selenium nanoparticle supplementation improves poultry products’ antioxidant defence and shelf life by reducing lipid peroxidation [115,122]. Selenium nanoparticle-enriched eggs have increased albumen quality, yolk integrity, and antioxidant potential, whereas selenium-fortified meat retains great colour, tenderness, and nutritional value [31,187,188]. Importantly, the utilization of SeNPs has dual benefits, optimizing poultry productivity and contributing to public health nutrition, without altering the organoleptic qualities of the final products. However, obtaining safe and effective selenium nanoparticle biofortification necessitates careful regulation of dose, nanoparticle size, and physicochemical form, as excessive accumulation may be toxic to both animals and consumers. Future research should focus on consistent safety assessments, kinetic analysis of selenium transfer, and determining human bio-accessibility from enhanced poultry products. Integrating nanotechnology-based micronutrient fortification into poultry production provides an innovative, long-term solution for addressing shortages of selenium while linking the feeding of animals with human health interests [189].

## 5. Limitations and Perspectives

Advances in nanotechnology have enabled the development of diverse nanoparticles with broad applications in poultry production, including rapid and precise disease diagnostics, enhanced growth performance in broilers and laying hens, immunomodulation, and antimicrobial activity through the suppression of pathogenic microorganisms. Despite these promising benefits, the potential safety and toxicity risks associated with nanoparticle supplementation must be thoroughly assessed to safeguard both poultry and human health prior to large-scale commercial adoption [189]. Furthermore, although nanoparticles of selenium—particularly red SeNPs—have shown significant advantages in antioxidant capacity, immune enhancement, and productivity, grey SeNPs remain considerably understudied. Their bioavailability, metabolic behaviour, long-term safety, and comparative efficacy in poultry production systems require further comprehensive investigation before they can be recommended for routine use in the poultry industry.

## 6. Conclusions

Selenium nanoparticles represent a promising advancement in poultry nutrition and health management, offering enhanced antioxidant protection, immune support, and production efficiency under both optimal and stress-inducing conditions. Their superior bioavailability and reduced toxicity compared to traditional selenium sources make them particularly advantageous for modern poultry systems facing environmental, nutritional, and biological stressors. SeNPs not only improve growth performance, feed efficiency, and reproductive outcomes but also contribute to the production of selenium-enriched meat and eggs with added functional value for human consumption. Moreover, SeNPs exhibit important biomedical properties, including anti-inflammatory, anticancer, antidiabetic, and dermatological benefits, further emphasizing their significance beyond animal nutrition. However, the fine line between selenium’s nutritional benefits and toxicological risks underscores the need for precise dosing and standardized regulations. Future studies should prioritize the exploration of underrepresented SeNP types (e.g., grey Se), long-term safety assessments, and the mechanisms governing selenium transfer to animal products and human consumers. The integration of selenium nanoparticles in poultry diets offers a sustainable and science-based approach to improving animal performance and public health, provided its application is guided by rigorous research and regulatory oversight.

## Figures and Tables

**Table 3:** Impact of selenium nanoparticles on poultry growth and production performance.

Birds	Impact of Selenium Nanoparticles Under Different Temperatures	Reference
Broiler	Enhances birds’ growth progression, increasing body weight, feed intake, and conversion.	[100]
	Improvement of antioxidant and antibacterial defence systems and reducing lipid accumulations for meat quality	[119]
	Decreasing mortality ratio, ameliorating antioxidant and immune systems under heat stress, and they are accustomed to warm conditions and increasing their production and growth	[86]
	Effective doses of Se-NPs ranged between 0.3 and 0.5 mg/kg, and the overdose is toxic and causes alterations in liver and macromolecule metabolisms, and it may promote bird death.	[31]
	Gives an antioxidant impact at 21 °C by improving animal health, reducing cholesterol levels in plasma, and augmenting HDL concentrations.	[120]
	Broiler chicks under 33–35 °C and selenium nanoparticles addition gave an anti-apoptotic effect.	[121]
	Biogenic SeNPs synthesized using *Bacillus subtilis* improve gut microbiota composition and immune responses under heat stress.	[104]
	SeNPs derived from Capsicum annuum improve carcass yield, blood biochemistry, immunity, and profitability in heat-stressed broilers.	[103]
	Biologically produced SeNPs boost growth, carcass traits, and economic returns in heat-challenged broilers.	[105]
	Chitosan-loaded SeNPs improve intestinal morphology, beneficial microflora, and nutrient absorption under thermal stress.	[106]
Hens	Improve digestive tube functions by reaching a beneficial microbial repertoire.	[117]
	Se-NPs support hen’s laying and reproduction performance with a low amount and controlled temperature.	[122]
	Se-NPs enhance laying hens’ production and the quality of their eggs under heat stress.	[123]
	Chitosan selenium nanoparticles enhance egg quality, shell strength, and selenium deposition in ageing hens under environmental stress.	[108]
	SeNPs improve laying rate, boost antioxidant resistance, and elevate selenium in liver and muscle tissue.	[109]
	Boosts egg selenium content and lipid stability, extending shelf life under temperature fluctuations.	[110]
	Strengthens immune function and improves laying performance under stress, with elevated yolk selenium.	[111]
Japanese quails	Enhanced its feed intake from 26.62 g to 26.63 g in 21 days, the birds gained weight, MDA levels were lower, GPx and TxR activities were at the highest levels, and the mortality rate was diminished compared with control samples.	[87]
	Quails supplied with Che-SeNPs gained weight, and antioxidant power was increased by SOD, GPx actions, and a remarkable increase in the levels of reduced glutathione GSH. At the same time, MDA concentrations measured and harmed microbial count in the birds were low, which supported immune functions and raised immunoglobulin production.	[69]

## Data Availability

No new data were created or analyzed in this study.

## Abbreviations

The following abbreviations are used in this manuscript:

SeNP	Selenium nanoparticles
GPx	Glutathione peroxidase
SOD	Superoxide dismutase
TAC	Total antioxidant activity
HSPs	Heat shock proteins
Se	Selenium
NADPH	Nicotinamide adenine dinucleotide phosphate
NAD	Nicotinamide adenine dinucleotide
KSCs	Keratinocyte Stem Cells
VEGF	vascular endothelial growth factor
ATP	Adenosine triphosphate
ADP	Adenosine diphosphate
UV	Ultraviolet radiation
DNA	Deoxyribonucleic acid
IL-6	Interleukin 6
TNF-α	Tumour necrosis factor
MsrB1	Methionine Sulfoxide Reductase B1
SelenoP	Selenoprotein P
SeP	Selenoprotein
TXNRD1	Thioredoxin Reductase 1
